# Learning Desire Is Predicted by Similar Neural Processing of Naturalistic Educational Materials

**DOI:** 10.1523/ENEURO.0083-19.2019

**Published:** 2019-10-02

**Authors:** Yi Zhu (朱怡), Yafeng Pan (潘亚峰), Yi Hu (胡谊)

**Affiliations:** 1 School of Psychology and Cognitive Science, East China Normal University, Shanghai 200062, People’s Republic of China; 2 Neuropsychology and Functional Neuroimaging Research Unit (UR2NF), ULB Neuroscience Institute (UNI), Université Libre de Bruxelles, 1050 Bruxelles, Belgium

**Keywords:** electroencephalography (EEG), intersubject correlation (ISC), learning desire, motivational effectiveness, naturalistic stimuli, neural similarity

## Abstract

Naturalistic stimuli can elicit highly similar brain activity across viewers. How do naturalistic educational materials engage human brains and evoke learning desire? Here, we presented 15 audiovisual course clips (each lasting ∼120 s) to university students and recorded their neural activity through electroencephalography. Upon finishing all the video viewings, subjects ranked 15 courses in order of learning desire and reported the reasons for high learning desire (i.e., “value” and “interest”). The brain activity during the video viewing was measured as the neural similarity via intersubject correlation (ISC), that is, correlation between each subject’s neural responses and those of others. Based on averaged learning desire rankings across subjects, course clips were classified with high versus medium versus low motivational effectiveness. We found that the ISC of high effective course clips was larger than that of low effective ones. The ISC difference (high vs low) was positively associated with subjects’ learning desire difference (high vs low). Such an association occurred when viewing time accumulated to ∼80 s. Moreover, ISC was correlated with “interest-based” rather than “value-based” learning desire. These findings advance our understanding of learning motivation via the neural similarity in the context of on-line education and provide potential neurophysiological suggestions for pedagogical practices.

## Significance Statement

This study shows that naturalistic educational materials with high motivational effectiveness elicit larger neural similarity across learners than less effective ones. Importantly, the neural similarity serves as a sensitive predictor of learners’ course-learning desire. It is suggested that the use of an emerging electroencephalography-derived intersubject correlation approach works with evaluating the quality of audiovisual educational materials. Hence, such a novel approach is promising to provide neurophysiological information for pedagogical practices.

## Introduction

Learning desire is an important prerequisite for human learning to occur. How to evoke learning desire is a persistent concern in the field of educational psychology and pedagogy (Todd, 2013). Recently, on-line courses have brought a tremendous transformation to education, as evidenced by their use in many open learning systems, such as Coursera and Khan Academy (Copley, 2007; Waldrop, 2013). Compared with the traditional classroom learners, on-line learners experience lower-level interactivity and thus are more susceptible to quitting learning or dropping out of courses (Szpunar et al., 2013; Kizilcec et al., 2015). Therefore, evoking learning desire is of great importance, especially in the context of on-line education (Visser, 1998; Keller and Suzuki, 2004). To this end, one good practice is to introduce audiovisual materials during the introductory phase of the course (Grant and Thornton, 2007; Kay, 2012).

Currently, there are two main hypotheses that account for potential factors contributing to learning desire. First, the “value-based” hypothesis (Atkinson, 1957; Eccles et al., 1983) proposes that helping learners perceive the value (e.g., utility value) will effectively promote learning desire. The perceived utility value of courses influences course enrollment decisions (Updegraff et al., 1996; Canning et al., 2018) and academic achievements (Hulleman et al., 2010). Second, the interest-based hypothesis (Krapp, 2002; Hidi and Renninger, 2006), postulates that guiding learners to develop interest will effectively boost learning desire. Interest, as the saying goes, is the best teacher. Interest contributes to learners’ further study (Mitchell, 1993; Ainley et al., 2002; Harackiewicz et al., 2002; Renninger and Hidi, 2002; Schiefele, 2009) and improves learning performance (Rotgans and Schmidt, 2014).

In the context of on-line education, learning desire evoked by audiovisual educational materials has been rarely studied from the neural perspective. To decode human brain activity during real-world experiences, previous studies have measured individuals’ neural responses to discrete and simplified artificial stimuli; these responses comprise electroencephalography (EEG)-derived event-related potentials and functional magnetic resonance imaging (fMRI)-derived blood oxygenation level-dependent (BOLD) signals (Spiers and Maguire, 2007). Beyond all that, emerging neuroscience research has started to measure the neural similarity (i.e., group-level similar neural responses) to concrete and complex naturalistic stimuli from a “collective-brain” perspective. Indeed, when exposed to the same stimulus, individual brains tend to tick collectively in synchronized spatiotemporal patterns (Hasson et al., 2004). The neural similarity can be quantified by intersubject correlation (ISC), that is, correlation between individual subject’s neural responses and those of others (Cohen et al., 2018). Using the ISC approach, previous fMRI research reveals that movie viewing elicits highly similar neural activity across viewers (Hasson et al., 2004). Within several-minute narratives, time-resolved ISC peaks during the viewing of scenes with high emotional arousal and valence (Hasson et al., 2004; Nummenmaa et al., 2012). Moreover, ISC is indicative of the powerfulness of political speeches (Schmälzle et al., 2015) and the effectiveness of antialcohol public service announcements (Imhof et al., 2017).

Apart from fMRI-derived ISC, previous EEG studies have captured significantly correlated components during the watching of movie clips, TV series, and commercials (Dmochowski et al., 2012, 2014). Correlated components were extracted from multichannel EEG time series to maximize the correlation based on a signal decomposition method (Dmochowski et al., 2012). EEG-derived ISC has been found to indicate attentional engagement during the narrative video viewing (Dmochowski et al., 2012; Cohen et al., 2017) and preferential attitudes toward Super Bowl commercials (Dmochowski et al., 2014). In a recent study, learners were asked to attentively or inattentively watch on-line educational videos, during which their brain activity was measured (Cohen et al., 2018). EEG-derived ISC discriminates between the attentive and inattentive viewings and predicts the learning performance. In a real-world classroom, EEG-derived ISC has also been found to associate with engagement and attentional modulation (Poulsen et al., 2017).

Building upon previous findings, the EEG-derived ISC approach holds the potential to uncover the neural underpinnings during the natural processing of audiovisual educational materials. In current study, we recorded EEG signals while learners were viewing audiovisual course clips. The ISC approach was adopted to examine the neural similarity across learners. Upon finishing all the video viewings, subjects ranked 15 courses in order of learning desire and reported the reasons of high learning desire (i.e., “value” and “interest”). The viewing of course clips was expected to prompt significant neural similarity across learners because brains tend to tick collectively during natural vision (Hasson et al., 2004; Dmochowski et al., 2012). Moreover, considering the potential links from the neural similarity to the effectiveness of naturalistic materials (Schmälzle et al., 2015; Imhof et al., 2017), and subjects’ attentional engagement (Dmochowski et al., 2012; Cohen et al., 2017; Poulsen et al., 2017) and preferential attitudes (Dmochowski et al., 2014), we expected that the neural similarity could be indicative of the motivational effectiveness of course clips and serve as a predictor of learning desire. Specifically, we hypothesized that (1) ISC should be higher for course clips ranked with high versus low learning desire, and (2) the ISC difference (high vs low) should be positively correlated with subjects’ learning desire difference (high vs low). Finally, to provide neurophysiological suggestions for why some naturalistic educational materials elicited high learning desire, we explored the association between ISC and potential reasons [e.g., value (Hulleman et al., 2008) and interest (Harackiewicz et al., 2002)].

## Materials and Methods

### Subjects

Fifteen subjects (three males; mean age, 21 years; age range, 18–25 years) were recruited through public announcement at the East China Normal University (Shanghai, People’s Republic of China). All of them were right handed and in good health, and had normal or corrected-to-normal vision and no history of neurological or psychiatric disorders. Monetary compensation was afforded for their participation. The study was approved by the Committee on Human Research Protection of East China Normal University (HR 064-2017). All subjects provided a written, signed informed consent form prior to the experiment.

### Materials

Fifteen courses from Massive Open Online Courses (http://www.icourse163.org) were selected based on the following three criteria: (1) being designed by National Key Universities to ensure the production quality; (2) covering various topics in humanities, social sciences, and natural sciences; and (3) on-line enrollments of those courses were various (see details in Table 1). We focused and selected the introductory parts of those several-hour on-line video courses (https://www.icourse163.org/course/WHU-85001), since the initial learning phase exerts an important effect on learning desire (Visser, 1998; Keller and Suzuki, 2004). The selected course clips were then edited (i.e., 1 s fade-out; resolution, 1280 × 720) using Movie Maker (Windows, Microsoft). The duration of each course clip lasted for ∼120 s (mean ± SD, 127 ± 41 s; range, 57–215 s).

**Table 1.** Summary of course clips

					Onlineenrollment	
No.	Course title	Topic	Duration (s)	URL-ending *	Mean	Rank
1.	Psychological Health of College Students	Psychology	100	NEU-1001930012#/info	2991	5
2.	Taoist Wisdom	Philosophy	81	XJTU-1001522001#/info	3528	2
3.	Chinese Poetry Art	Literature	149	SCU-21006#/info	4937	1
4.	Silk Culture and Products	Art	129	SUDA-1001754250#/info	160	15
5.	Managerial Communication	Management	133	NUEPU-292001#/info	2168	6
6.	Economic Geography and Vicissitude of Enterprises	Economics	67	ZNUEDU-1001615011#/info	848	10
7.	Culture of Mathematics	Math	123	NANKAI-312001#/info	3350	4
8.	Applied Optics	Physics	57	BIT-1001606003#/info	1481	9
9.	Medicinal Chemistry	Chemistry	121	CPU-1001570004#/info	1831	7
10.	Engineering Materials and Manufacturing	Engineering	133	SDU-306001#/info	776	12
11.	Cytobiology	Biology	132	SCU-46011#/info	1511	8
12.	First Aid General Knowledge	Medical Science	170	WHU-85001#/info	3368	3
13.	Space Humanities and Arts	Interdiscipline	215	NUAA-1001764004#/info	836	11
14.	Medical Ethics	Interdiscipline	170	XJTU-47022#/info	382	13
15.	Fantastic Bionics	Interdiscipline	129	JLU-32007#/info	187	14

On-line enrollment (person-time/session) was recorded by the date of 2017/03/26.

*URL beginning with http://www.icourse163.org/course/.

### Procedures

**Figure 1.**
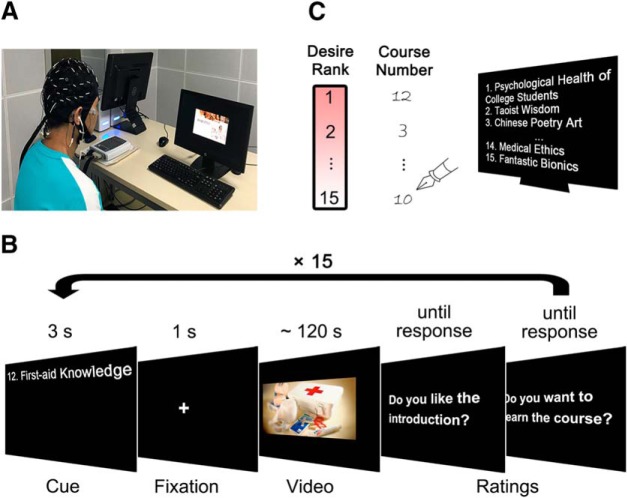
Schematic illustration of the experimental procedure. ***A***, Experimental setup. ***B***, Events and time flows in a trial. ***C***, Subjects ranked courses based on their learning desire form 1 (highest) to 15 (lowest). Note that in the following analyses, rankings were reversely coded.

During the experiment, subjects were individually seated in front of a 19.5 inch monitor in an electromagnetic-sound-shielded room, and wore earphones and an EEG recording cap (Fig. 1*A*).

There were 15 trials corresponding to 15 course clips. One trial entailed the following steps. First, a course title together with its preassigned course number (Table 1, see details) appeared for 3 s, followed by a 1 s fixation. Next, subjects watched a course clip. After that, subjects provided answers to “Do you like the introduction?” and “Do you want to learn the course?” (1–100, from “not at all” to “very much,” until response; Fig. 1*B*). Controlled by E-prime software (version 2.0; Psychology Software Tools), the presentation order of trials (course clips) was randomized across subjects.

Upon finishing 15 trials, 15 course titles with their course numbers were presented together on the screen. Subjects were then instructed to rank the 15 courses in order of their learning desire from 1 (most) to 15 (least). To do so, subjects wrote down corresponding course numbers beside a column of rankings (1–15) using a paper and pen (Fig. 1*C*). Upon finishing the course ranking, subjects were asked to rate the potential reasons to which they attributed their high learning desire on a 4-point scale from 1 (strongly disagree) to 4 (strongly agree). Two items testing the most concerned reasons, value and interest, were included: “learning the introduced course is useful for me” (Hulleman et al., 2010) and “I am interested in the introduced course” (Nuutila et al., 2018). As suggested by the precollected data from independent raters (see Statistical analyses), subjects reported reasons only for their top two courses (i.e., those were ranked with 1 and 2). To note, learning desire rankings of courses were later coded in reverse (i.e., 16 minus original rank), such that larger rankings indicated higher course-learning desire.

### EEG data acquisition and preprocessing

Brain signals were recorded via a 64-channel EEG apparatus (Compumedics NeuroScan), in accordance with the international 10/10 system. The electrooculograms (EOGs) were recorded via four auxiliary electrodes. Two horizontal electrodes were placed lateralized to two eyes, while the other two vertical electrodes were placed over the upper and lower sides of the left eye. Data collected from the two horizontal electrodes and the two vertical electrodes were synthesized respectively and merged into one horizontal channel and one vertical channel. Impedances were kept to <10 kΩ. Signals were digitized at a sampling rate of 1000 Hz.

Following the study by Dmochowski et al. (2012), preprocessing of EEG data was performed using custom MATLAB (R2016b, MathWorks) scripts with EEGLAB toolbox (version 14.1.0; Delorme and Makeig, 2004). Data were filtered at a 1 Hz high-pass and a 50 Hz notch, and downsampled to 250 Hz. As we focused on the EEG activity during the watching of course clips, data were segmented from the beginning to the end of each video. Eye-movement artifacts were corrected using a regression-based approach (Gratton et al., 1983; Elbert et al., 1985), as follows: (1) approximating the amplitude relation between EOG channels and each EEG channel and (2) then subtracting the estimated proportion of the EOG from the EEG. The regression-based correction was separately performed on the entire data block corresponding to each video. Two EOG channels and two mastoid channels were then omitted, leaving 60 channels in the following analyses. Bad channels were automatically rejected for exceeding mean channel power by 5 SDs. Outlier samples in each kept channel were rejected for their magnitudes exceeding the mean of that channel by more than 5 SDs. Data within −40 to +40 ms (20 sampling points) relative to each identified artifactual outlier were additionally rejected, and all were replaced by zeros. The preprocessed EEG data entered into subsequent analyses.

### Intersubject correlation

The analysis of ISC (Dmochowski et al., 2012, 2014; see more details at www.parralab.org/isc/) was computed to quantify the neural similarity while subjects were watching the same naturalistic stimuli. It aims to find components (here, linear combinations of electrodes) capturing maximal correlation across all subjects. The concept of maximizing correlations resembled that in canonical correlation analysis (Hotelling, 1936), with a constraint being that the same projection vectors were used for all the datasets.

**Figure 2.**
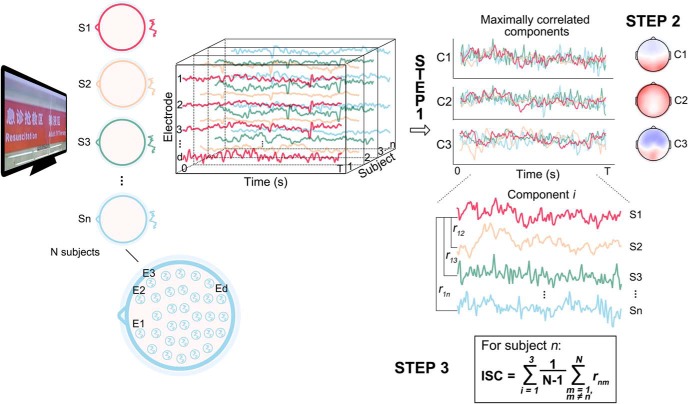
Overview of the three-step ISC analysis. Neural responses are recorded on *D* electrodes from *N* subjects during the time (0–*T* s) of stimuli presentation. First, a few (first three in this study) maximally correlated components are extracted. Second, the spatial distribution of each component is visualized. Third, for each subject, ISC is measured as the sum of the averaged correlation coefficients between that subject and remaining subjects over the first three components. Ed, Electrode *d*; Sn, subject *n*. Ci, component *i*.

ISC analysis was performed individually for each course clip. It included three steps (Fig. 2). First, correlated components were captured across all subjects’ neural datasets (subjects × electrodes × time-points) by solving an eigenvalue problem similar to the principle component analysis (Parra and Sajda, 2003). Second, the three strongest correlated components (i.e., C1, C2, and C3) were retained while other smaller ones were ignored (Dmochowski et al., 2012, 2014; Ki et al., 2016). Spatial distributions of the C1, C2, and C3, informing the approximate locations of the underlying neuronal sources, were visualized (Parra et al., 2005; Haufe et al., 2014). Finally, for each subject, component-wise (i.e., projected EEG data) correlations were computed between this subject and each of all remaining subjects, and then averaged. The ISC was then obtained as the sum of the correlation coefficients over C1, C2, and C3.

### Statistical analyses

Following previous EEG-ISC studies (Dmochowski et al., 2012; Ki et al., 2016), we used a phase randomization technique to determine the chance-level ISC of each course clip (Theiler et al., 1992). To do so, fast Fourier transformation was first used to extract the original phases and amplitudes of preprocessed EEG data. Then, randomly generated phases were added to the original phases. With unchanged original amplitudes, inverse fast Fourier transformation was then used to reconstruct phase-randomized surrogate EEG data. By this step, the phase-randomized EEG surrogate data were not supposed to correlate across subjects. For each course clip, the significance level was determined by comparing the ISC of the original data to the mean ISC of 1000 phase-randomized surrogate datasets. The resulting *p* values for 15 course clips were then corrected using the false discovery rate (FDR) procedure (Benjamini and Yekutieli, 2001).

We calculated the motivational effectiveness of course clips by averaging the learning desire rankings across subjects. Fifteen course clips were then classified into three categories with different degrees of motivational effectiveness, high (average rankings from 11 to 15) versus medium (6–10) versus low (1–5); two clips (i.e., no. 3 and no. 12) were classified into the high effective category, 2 (i.e., no. 10 and no. 13) into the low effective category, and 11 (i.e., the remaining) into the medium effective category. Such a classification was validated by an additional group of independent raters. Prior to the EEG experiment, using the identical experimental procedures except for the EEG collection, behavioral data were precollected from an independent group of 25 subjects (six males; mean age, 21 years; age range, 19–25 years; 1 subject was left handed). The independent raters classified exactly the same 2 clips into the high effective category, exactly the same 2 into the low effective category, and exactly the same remaining 11 into the medium effective category as the EEG group did. To validate the use of group-averaged rankings for classification, we measured the variability of learning desire rankings for course clips across subjects (including EEG subjects and independent raters) using intraclass correlation (ICC). The ICC reached 0.93, suggesting that the variability of the group-averaged rankings for course clips across subjects were fairly low (Koo and Li, 2016).

With the aforementioned classification of course clips, we then conducted one-way repeated-measures ANOVAs to relating motivational effectiveness (high vs medium vs low) with ISC values (i.e., ISC and subcomponents). Specifically, for each subject, ISC values of each effective category were first averaged across course clips in that category and then compared using repeated-measures ANOVAs, with motivational effectiveness (high vs medium vs low) as a within-subject variable. For *post hoc* pairwise comparisons, we used paired *t* tests with FDR correction.

We further conducted a Pearson correlation analysis between ISC difference (high vs low) and learning desire difference (high vs low). Difference values (i.e., ISC difference and learning desire difference) were calculated by subtracting values of the low effective category (after averaging values across two involved course clips) from values of the high effective category (after averaging values across two involved course clips). To note, considering that ISC varies due to individual differences (Petroni et al., 2018), we chose to use the difference values rather than values of either low or high effective category. The ISC of the low effective category served as an active baseline and was subtracted to control for individual differences. Here we focused on learning desire rankings decided after all the viewings of course clips rather than learning desire ratings of “do you want to learn the course?” collected after each course clip viewing since they were highly correlated with each other (*r*_(15)_ = 0.97, *p* < 0.001) and the former were less biased to limited information. Given the evidence linking subcomponents of ISC (i.e., C1, C2, and C3) to separate cognitive functions (Dmochowski et al., 2014; Cohen and Parra, 2016), parallel correlation analyses were also separately performed between subcomponent ISCs difference (high vs low) and learning desire difference (high vs low).

Previous studies have found that human brain is optimized to make the fastest decision at a specified accuracy after successively integrating external perceptional inputs (Bogacz et al., 2006; Gold and Shadlen, 2007; de Gardelle and Summerfield, 2011; Tsetsos et al., 2012). How early brain responses predict subsequent behaviors has been computed by identifying the earliest time-point at which time-cumulative brain activity was significantly correlated with subsequent behaviors (Jiang et al., 2015; Zheng et al., 2018; Liu et al., 2019). Accordingly, we explored how early ISC predicted learning desire by identifying the earliest time-point, at which time the cumulative ISC difference (high vs low) was correlated with subsequent learning desire difference (high vs low). Specifically, time course correlation analyses between time-cumulative ISC difference and subsequent learning desire difference were repeatedly performed from 0.1 to 133 s (the shortest duration among the four course clips involved in low and high effective categories) with a time increment of 0.1 s. The time-cumulative ISC at a certain cumulative time (*ct*) was computed by the time points from 1 to *ct* × 250 (sampling rate). The subsequent learning desire difference used in the time course correlation analyses was same as that used in the aforementioned full time correlation analysis. The resulting *p* values, at the same size of repeated times for correlation analyses, were then corrected using FDR methods. Accordingly, if there existed a certain time point after which *p* values of the correlations between the time-cumulative ISC difference and learning desire difference started and were maintained to survive the FDR correction, such a time-point would be labeled as the starting time point that ISC could successfully predict learning desire. Time increments of 0.5, 1, 2, 5, and 10 s also returned similar results. In addition, parallel time course correlation analyses were performed separately for subcomponent ISCs (C1, C2, and C3).

Moreover, we attempted to provide neurophysiological suggestions for why some naturalistic educational materials elicited high learning desire. To do so, for individual subjects, we focused on reason ratings (i.e., value and interest) and ISC of their own top two course clips with the highest rankings. Value and interest ratings were averaged across individual-level top two course clips and compared using a paired *t* test. Next, we conducted Spearman correlation analyses between reason ratings (i.e., interest and value) and ISC, which was also averaged across individual-level top two course clips for each subject.

### Code accessibility

The code described in the article is freely available on-line at https://github.com/YiZhuECNU/EEG-ISC.git. The code is available as Extended Data 1. It can be performed using MATLAB (version 2016b) in a Windows 10 system.

10.1523/ENEURO.0083-19.2019.ed1 Extended Data 1 This is the MATLAB code to compute EEG-derived ISC using correlated component analysis, specified for EEG data collected from the Compumedics NeuroScan system. You will need EEGLAB (version 14.1.0) and Curry7 format EEG data to run Step1_preprocess_demo.m. You will need the following files to run Step2_ISC_demo.m: (1) runisc.m (EEG-ISC specific code); (2) topoplot.m (stand-alone version of the EEGLAB popular display function); (3) Neuroscan64.loc (Neuroscan location file for topoplot); (4) notBoxPlot.m (stand-alone version of Rob Campbell’s scatter plot); (5) Data file (e.g., v12. mat, EEG data with 60 electrodes from 15 subjects while watching the course clip No. 12, time-points × channels × subjects). Download Extended Data 1, ZIP file.

## Results

### The significant ISCs for course clips

**Figure 3.**
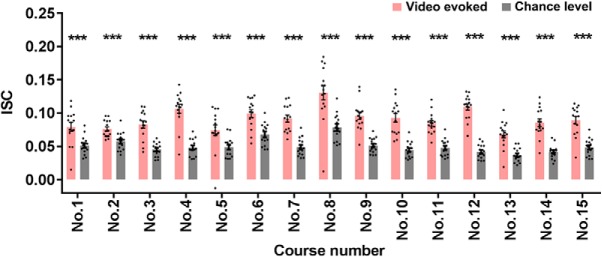
Video-evoked versus chance-level ISC of each course clip. ISC evoked by each course clip significantly exceeded its chance level. Each dot represents one subject. Error bars indicate SEs. ***p < 0.001, FDR corrected.

As expected, each of 15 video-evoked ISCs (i.e., the averaged ISC across all the subjects) significantly exceeded its corresponding chance-level ISC determined by phase-randomized surrogated data (*p* values < 0.001, FDR corrected; Fig. 3), indicating that course clips induced a significant learner-wise similar neural activities.

### ISC of high versus medium versus low effective course clips

**Figure 4.**
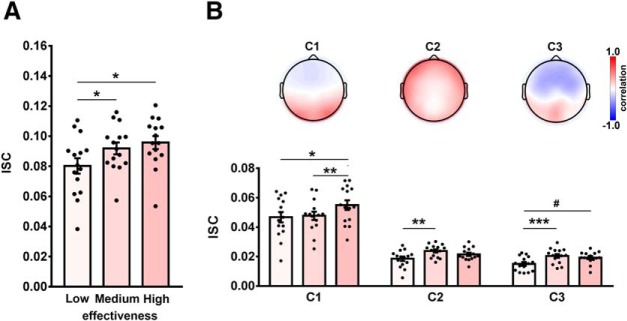
ISC of high versus medium versus low effective course clips. ***A***, ISCs of high and medium effective clips were respectively larger than that of low ones. ***B***, Top, One representative illustration (i.e., the highest effective course clip) of the scalp projections of the first three maximally correlated components (i.e., C1, C2, and C3). Color indicates how strongly the component activity correlated with the EEG signals recorded on different electrodes across the scalp. Bottom, Subcomponent ISCs were also enhanced when the motivational effectiveness of course clips increased. Each dot represents one subject. Error bars indicate SEs. #*p* < 0.1, *p < 0.05, **p < 0.01, ***p < 0.001, FDR corrected.

We next sought to identify whether ISC varied by motivational effectiveness. A one-way repeated-measures ANOVA comparing the ISC across motivational effectiveness (high vs medium vs low) factor on ISC was conducted. Results revealed a significant main effect (*F*_(2,28)_ = 8.36, *p* = 0.001, *η*_p_^2^ = 0.37). Follow-up pairwise comparisons showed that the ISC was significantly larger for the medium effective category (mean ± SD, 0.09 ± 0.02; *t*_(14)_ = 3.18; corrected *p* < 0.05; Cohen’s *d* = 0.68) and for the high effective category (0.10 ± 0.02; *t*_(14)_ = 3.25; corrected *p* < 0.05; Cohen’s *d* = 0.86) than that for the low effective category (0.08 ± 0.02; Fig. 4*A*).

Similar analyses on subcomponent ISCs (C1, C2, and C3) consistently showed the main effect of motivational effectiveness (*F* values > 6.78; *p* values < 0.004; *η*_p_^2^ > 0.33). Follow-up pairwise comparisons showed the following results: for C1, the ISC of the high effective category was larger than that of the medium effective category (*t*_(14)_ = 3.98; *p* < 0.01; Cohen’s *d* = 0.58) and that of the low effective category (*t*_(14)_ = 3.03; *p* < 0.05; Cohen’s *d* = 0.59); for C2, the ISC of the medium effective category was significantly larger than that of the low effective category (*t*_(14)_ = 4.00; *p* < 0.01; Cohen’s *d* = 0.98); for C3, the respective ISC of the medium and high effective categories was larger (*t*_(14)_ = 6.28; *p* < 0.001; Cohen’s *d* = 1.01) and tended to be larger than that of the low effective category (*t*_(14)_ = 2.58; *p* < 0.1; Cohen’s *d* = 0.89; Fig. 4*B*, bottom).

Representative spatial projections of three correlation-maximizing components on the scalp showed that the first component was symmetric and marked approximately in the frontal and occipital lobes, the second component was approximately in the bilateral frontotemporal lobes, and the third component was marked widely in the parietal cortex (Fig. 4*B*, top). Such scalp projections resulted from viewing course videos that were in accordance with those previously found for other audiovisual stimuli (Dmochowski et al., 2012, 2014).

### ISC as a predictor of course-learning desire

**Figure 5.**
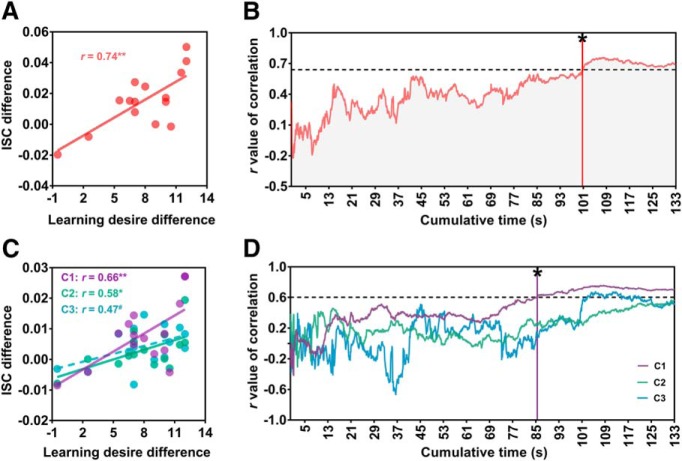
ISC predicted course-learning desire. ***A***, Pearson correlation indicated ISC difference (high vs low) positively associated with the subject’s learning desire difference (high vs low; *r*_(15)_ = 0.74, *p* = 0.002). ***B***, ISC difference became a significant correlate of learning desire difference after ∼100 s of watching. The vertical red line with an asterisk indicates the earliest time (100.6 s) at which such a correlation reached the significance. The horizontal dashed line indicates the correlation coefficient (*r*_(15)_ = 0.64, *p* < 0.05, FDR corrected). ***C***, Pearson correlations indicated differences in subcomponent ISCs independently associated with the subject’s learning desire difference. ***D***, For C1, the vertical purple line with an asterisk indicates the earliest time (85.5 s) at which correlation reached the significance. The horizontal dashed line indicates the correlation coefficient (*r*_(15)_ = 0.60, *p* < 0.05, FDR corrected). C2 or C3 showed no such early prediction effect. Each dot represents one subject. #*p* < 0.1, *p < 0.05, **p < 0.01.

We then tested whether the ISC predicted subjects’ course-learning desire. ISC difference (high vs low) was significantly correlated with learning desire difference (high vs low: *r*_(15)_ = 0.74, *p* = 0.002; Fig. 5*A*). Parallel correlation analyses for subcomponent ISCs found that the difference in subcomponent ISCs (high vs low) was independently correlated with the learning desire difference (high vs low: C1, *r*_(15)_ = 0.66, *p* = 0.007; C2, *r*_(15)_ = 0.58, *p* = 0.02; C3, *r*_(15)_ = 0.47, *p* = 0.08; Fig. 5*C*).

To identify how an early ISC predicted learning desire, time course correlation analyses were repeatedly conducted with a 0.1 s time increment from 0.1 to 133 s between time-cumulative ISC difference (high vs low) and subsequent learning desire difference (high vs low). We found that at 100.6 s after the video onset, the time-cumulative ISC difference (high vs low) started to become a significant predictor of subsequent learning desire difference (high vs low: *p* < 0.05, FDR corrected; Fig. 5*B*). Later on, correlations remained constantly significant until the video ended. Parallel time course correlation analyses were conducted for each subcomponent ISC. Results revealed a key role of C1 (but not of C2 and C3) in the prediction (starting at 85.5 s; Fig. 5*D*).

### The association between ISC and interest/value

**Figure 6.**
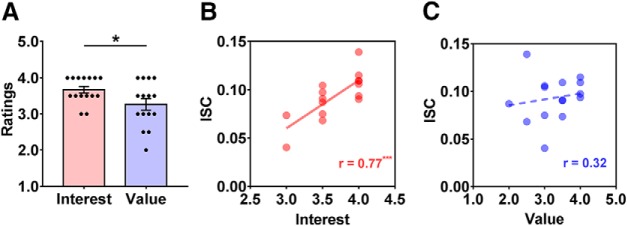
ISC was associated with interest rather than value. ***A***, Ratings of interest significantly exceeded those of value. ***B***, ***C***, For individual-level high effective course clips, the Spearman correlation indicated that ISC positively associated with the ratings of value (*r*_(15)_ = 0.77, *p* = 0.0008; ***B***), but not with ratings of interest (*r*_(15)_ = 0.32, *p* = 0.25; ***C***). Each dot represents one subject. Error bars indicate SEs. *p < 0.05, ***p < 0.001.

In an attempt to provide neurophysiological suggestions for why some course clips are effective to evoke learning desire, we tested whether ISC was associated with value and/or interest. Behaviorally, ratings of interest (mean ± SD, 3.67 ± 0.36) were highest among all the reasons and significantly exceeded ratings of value (3.27 ± 0.62; *t*_(14)_ = 2.45, *p* = 0.03, Cohen’s *d* = 0.79; Fig. 6*A*). Moreover, the ISC for individual-level top two course clips was significantly correlated with ratings of interest (*r*_(15)_ = 0.77, *p* = 0.0008; Fig. 6*B*), but not of value (*r*_(15)_ = 0.32, *p* = 0.25; Fig. 6*C*).

## Discussion

Here we used an EEG-derived ISC approach to capture the degree of shared brain responses to naturalistic educational materials. Results revealed that (1) on-line course videos prompted similar neural activity across learners; (2) the neural similarity was enhanced by the motivational effectiveness of course clips for evoking learning desire; (3) the neural similarity was predictive of course-learning desire (even before finishing the viewing of the entire video); and (4) the neural similarity was associated with interest-based (rather than value-based) learning desire. These results are discussed successively as follows.

First, using EEG, we recorded learners’ general patterns of neuronal activity at the timescale during the watching of on-line course videos. We found that all 15 course clips, regardless of their motivational effectiveness for evoking learning desire, elicited significant neural similarity across learners. This result aligns well with pervious findings that brains of different individuals tend to act in unison during the natural watching (Hasson et al., 2004; Dmochowski et al., 2012, 2014; Ki et al., 2016). Thus, an ISC across multiple brains tends to provide a sensitive and quantifiable measure of the continuous neural responses to naturalistic stimuli. Critically, this measure makes it feasible for conventional laboratory paradigms to move beyond the rigid trial-based structure where discrete stimuli are repetitively presented.

Second, although all course clips prompted similar neural processing across learners, we found significantly larger ISC for high (vs medium, vs low) effective course clips. It seems that course clips, which engage learners’ brains more collectively, are more effective to evoke course-learning desire. This finding is consistent with prior studies demonstrating larger ISC during strong (vs weak) powerful political speeches (Schmälzle et al., 2015), and more (vs less) effective antialcohol public service announcements (Imhof et al., 2017). An ISC has also been found to predict the preferential effectiveness of advertisements in an EEG study (Dmochowski et al., 2014). However, here we failed to demonstrate that ISC scaled monotonically with the motivational effectiveness of course clips. To note, the duration of advertisements used in the study by Dmochowski et al. (2014) is identical (i.e., 30 s), while the duration of course clips used in our study is not (i.e., 57–215 s). We suspected that the longer watching of materials might damage the sustained attention or vigilance (Nuechterlein et al., 1983; Sarter et al., 2001), thence modulating the ISC (Ki et al., 2016; Iotzov et al., 2017; Cohen et al., 2018).

Third, course-learning desire could be predicted by the neural similarity. Moreover, time course analyses showed that ISC was predictive of subsequent course-learning desire after ∼80 s of watching of videos. The first maximally correlated component (C1) played a key role in such a prediction. Representative scalp projection of the C1 exhibited a symmetric pattern marked in the frontal and occipital electrodes. Such a component captures the neural activity possibly reflecting the visual processing (Dmochowski et al., 2012, 2014), suggesting that the visual property of educational materials is crucial for promoting learning desire. This finding is in accordance with those of previous studies showing that visual properties, such as saliency, influence the final decision (Milosavljevic et al., 2012; Towal et al., 2013). The second component (C2) in the bilateral frontotemporal lobes was possibly recruited in the auditory processing (Hickok and Poeppel, 2007). Besides, C1 and C2 might also capture supramodal responses (Cohen and Parra, 2016). The third component (C3) was marked widely in the parietal cortex, which might be associated with attentional modulation to audiovisual stimuli (Shomstein and Yantis, 2006; Nardo et al., 2011). The global scalp patterns observed in the current study aligned with those found in a previous fMRI study, where spatial dimension of the observed EEG-derived neural similarity was probed by regressing BOLD activation time series onto the neural similarity scores (Dmochowski et al., 2014). In a final detail, ∼80 s of video watching was sufficient to predict the course-learning desire. This finding bolsters the optimization of brain to make the fastest decision at a specified accuracy after successively integrating external perceptional inputs (Bogacz et al., 2006; Gold and Shadlen, 2007; de Gardelle and Summerfield, 2011; Tsetsos et al., 2012).

Fourth, we provided neurophysiological suggestions for why some course clips were effective to evoke learning desire by testing the association between ISC and value/interest. For course clips ranked with higher learning desire by individuals, (1) interest was reported to be a more important reason for further course study, and (2) neural similarity during the processing of those videos was associated with self-reported interest rather than value. These findings support interest-based learning desire hypothesis—learners’ interests effectively promote learning desire (Ainley et al., 2002; Harackiewicz et al., 2002; Krapp, 2002; Hidi and Renninger, 2006). Given the evidence that ISC is strongly modulated by attention (Ki et al., 2016; Iotzov et al., 2017; Cohen et al., 2018) and the potential association between attention and interest (Shirey, 1992), we suspected that attention might play a role in the relationship between ISC and interest-based learning desire. As an important note, although value-based learning desire hypothesis did not gain the supporting results in the current study, we could not assuredly draw a conclusion that value played no role in promoting learning desire. It might be the case that our ISC measure was not so sensitive to the value-based on-line learning. Future independent replications are needed to provide more evidence.

Several limitations of this work, along with future directions, deserve to be noted. First, scalp projections of correlated components are not valid to exactly locate brain sources due to the inherently limited spatial resolution of EEG. Therefore, future studies should consider adopting source analyses (e.g., standardized low-resolution brain electromagnetic tomography; Pascual-Marqui, 2002) with high-resolution EEG, as well as fMRI/MEG with satisfactory spatial resolution (Dmochowski et al., 2014). Second, *post hoc* power analyses with G*Power (Erdfelder et al., 1996) indicated that a sample size of ∼13 would be sufficient to obtain statistical power at the recommended 0.8 level (Cohen, 1988) with Cohen’s *d* = 0.86 reported in the result that ISC was larger for high (vs low) effective course clips. However, our sample size (*N* = 15) was far from adequate to examine how individuals’ factors (e.g., goal orientation; Elliot and Mcgregor, 2001) influenced the neural responses to educational messages in a top-down manner, calling for future studies. Finally, value and interest were viewed independently in the present study, since the correlation conducted on individual subjects between value and interest ratings for their own high effective course clips was not significant (*r*_(15)_ = 0.26, *p* = 0.34). However, value and interest have been found to interact with each other and have interplay effects on competence belief, success expectancy, and learning performance (Hidi and Renninger, 2006; Fryer and Ainley, 2018; Nuutila et al., 2018). Future studies might test the interplay of value and interest in other contexts of on-line learning [e.g., courses with (1) high value + high interest, (2) high value + low interest, (3) low value + high interest, and (4) low value + low interest, and the power of ISC measure to differentiate between them].

To sum up, the current results indicate that naturalistic educational materials with greater motivational effectiveness enhanced neural similarity across learners. Such enhanced neural similarity is predictive of learning desire, which is based on interest. From a collective-brain perspective, the use of EEG-derived ISC approach holds the potential to evaluate the motivational effectiveness of naturalistic educational materials. Our study paves the way to investigate learners’ motivation at a neurophysiological level in the context of on-line learning. It also holds relevance for instructional designs to aid learning interest deficit.

## Synthesis

Reviewing Editor: Satu Palva, University of Helsinki Decisions are customarily a result of the Reviewing Editor and the peer reviewers coming together and discussing their recommendations until a consensus is reached. When revisions are invited, a fact-based synthesis statement explaining their decision and outlining what is needed to prepare a revision will be listed below. The following reviewer(s) agreed to reveal their identity: Guillaume Dumas, Lucas Parra.

Synthesis

The study provides an interesting extension of previous works with inter-subject correlation, and it addresses a contemporary debate between “value-based” and “interest-based” hypotheses of learning motivation. More specifically, this paper discusses how the correlation across neural responses to educational videos, or inter-subject correlation (ISC) can be used to predict the desire to learn evoked by these videos. Both learning desire and ISC are assessed. Learning desire is also attributed to either interest or value of the videos. Overall, the manuscript is clearly written and interesting. However, the paper has several major issues that should be corrected before publication. These are detailed in below.

Major

The major problem in the manuscript was that the methods, data-analysis approaches or statistical analyses were not carefully explained and therefore the validity of the manuscript was difficult to evaluate. These problems are detailed below.

Although the manuscript was generally well written, the abstract was difficult to follow and would benefit from rewriting. Current findings should also be better integrated with previous work, specifically work using ISC and educational stimuli. It should also be better explained as to why ISC is thought to be a marker of educational desire. Furthermore, while an interpretation of C1 is provided in the discussion, C2 and C3 remained undescribed. It would be great to have at least a couple of sentences describing what those components could reflect at the cognitive level. There are also many grammatical errors in the manuscript. Please proof-read the manuscript carefully before submitting the revision.

Statistical analyses

it is stated that a correlation is computed between ISC and learning desire change. It is unclear how “learning desire change” is computed. Furthermore, it is stated that the ISC values for the low-effective videos were subtracted, would this not remove the difference between low and high effective videos that is meant to be tested. A better description of the statistical procedures and tests is necessary to make this assessment.

Methods

- How the authors are “regressing out the EOG channels”

- What consists the procedure of “phase-randomized surrogate data”.

- To create a null distribution for ISC, EEG was phase randomized. A more intuitive null distribution might be created by correlating the responses to one video with the responses to another and seeing what is the level of correlation when the assignment of the video is randomized. Would a null distribution such as this yield a similar result?

- It is stated that the present study will differentiate between “value” and “interest” based desire to learn, however the hypothesis as to why and how this might be the case are unclear. Is ISC expected to differ between the two, and if so, why?

- Were just the introductory parts of the online videos selected? What is meant by “intercepted the introductory parts”?

- How long were the original videos that excerpts were taken from?

- It is unclear what “course number” means in figure 1C

- The order of the experiment in Figure 1B is different from how it is described in the text. The question of “Do you want to learn the course?” is depicted as appearing after the clip in the figure, and before the clip in the text. Which one is it?

- Table S3 is referred to, but it is not included in the manuscript.

- How were the electrooculograms (EOG) recorded? How many EOG electrodes were used? Where were they placed?

- In the ISC calculation, were the components extracted for each video individually, or across all videos together?

- It is unclear if the ranking of course motivational effectiveness was done by averaging the rankings across subjects. It is stated that “mean ranks” were calculated. Were these the means across subjects?

- It is stated that additional raters were asked the effectiveness of the clips. What is meant by the “the classification of the course excerpts was agreed”? Did they have the same mean rankings? It seems like this is very unlikely, given that 15 items can be ordered in many different ways by chance (15 factorial).

- How was a within-subject factor of course excerpts created? This is unclear.

- It is stated that to examine the association between ISC and course learning desire, a correlation was computed between ISC change and learning desire change. What changed? I think what is meant by change is difference between videos, but I am not sure. Also, if the ISC values for the low effective courses were subtracted here (as is stated), wouldn't this negate the difference between high and low, and thus be simply a measure of just the ISC for the courses with high ISC?

- Why were separate correlation analyses performed for each of the sub-components (C1, C2, and C3)? What is the justification for doing both this and the summed ISC?

- The time cumulative ISC measure is an interesting idea, but how do you know that it is predictive of when ISC is predictive of learning desire?

- It is possible that ISC is a noisy predictor in such small time increments (0.1 second intervals). Did you do anything to ensure the robustness of a predictor that was trained over such brief intervals?

- Why was a paired t-test was performed to compare the difference between value and interest? Was it within subjects, or within videos?

- It should be explained how you calculated high vs medium vs. low effective. It is still not exactly clear how this was calculated, although it was probably done by looking at the mean across subjects. Could you please make this clearer?

- - Was the ANOVA done by combining videos from these different categories? Based on Figure 4 this seems to be the case. This should also be made clear in the text.

Also Results lack some details that are detailed below together with other suggestions.

- Bar plots can be misleading. This it is recommended that data in Figure 3 and Figure 6A should be presented like in Figure 4.

- As stated in the discussion, the overall study has only 15 excerpts and 15 participants. It would be important to report a power analysis to assess the robustness of the statistical analyses.

- Some videos appear to be closer to chance-level ISC than others (Figure 3). This seems potentially interesting. Is there any reason for this? Text

- - It is unclear what is correlated in Figure 5A. What is “ISC increase” what is “desire rank increase.” ISC increase may be the difference in ISC between low-ranked and high-ranked videos for that subject. Is it?

- How was desire rank increase calculated in the time course ISC correlation? Were subjects asked to dynamically rate how much they desired to learn about the topic as the video played? This is not stated in the methods section.

- How were ratings of interest and value calculated for the comparisons in section 3.4? Were the ratings averaged across subjects for the comparison in Figure 6A? And across movies for the comparison in Figure 6B? This should be made clear in the text.

Minor

- ISC code is on a dedicated webpage but custom codes for this paper are not. It would be nice to have them on a public repository to allow a better reproducibility of the results presented here.

- ISC in the introduction should be defined.

- ISC has been shown to be predictive of engagement by Cohen, Henin, & Parra, 2017 (a citation mentioned in the text for other reasons). It seems like it would be relevant to refer to this citation in the context of learning desire.

- The authors state that they were not able to replicate the results in Dmochowski et al 2014 because the duration of their stimuli varied across videos. Is it possible that they would have a more sensitive measure if they only used the first 30 seconds of each video, thereby constraining the duration of the ISC calculated from each video to be the same?

- - The authors state that the first component of ISC is related to visual processing. However, Cohen and Parra 2016 found this component to be “supramodal” or impervious to stimulus modality. The authors should reconsider their findings in light of this research.

- It was found behaviorally that interest is most predictive of learning desire, given that this is already known, what, if anything does the measure of ISC add?

- A stated limitation of the study is that EEG cannot be used to assess the sources of the component activity. However, Dmochowski et al 2014 used fMRI to localize this component activity. The authors should note this finding and speak about their results in light of the brain areas implicated in EEG-ISC found in this paper.

- What is meant by the third study limitation.

- How might future studies address the interplay between value and interest as motivators for learning desire? It seems like prior to the fourth study limitation value and interest were viewed as independent actors. How and in what ways do they interact?

- The idea of the “collective-brain” perspective is only introduced in the discussion. Please define this term and introduce it earlier in the paper.

A couple of grammar/style suggestions in the abstract:

“the naturalistic educational materials” -&gt; “naturalistic educational materials”

“Neural similarity” -&gt; “The neural similarity”

“for the further study” -&gt; “for further study”
